# Nanocrystalline Hydroxyapatite/Si Coating by Mechanical Alloying Technique

**DOI:** 10.1155/2012/390104

**Published:** 2012-01-23

**Authors:** Ahmed E. Hannora, Alexander S. Mukasyan, Zulkhair A. Mansurov

**Affiliations:** ^1^Faculty of Petroleum and Mining Engineering, Suez Canal University, Suez 43721, Egypt; ^2^Department of Chemical and Biomolecular Engineering, University of Notre Dame, Notre Dame, IN 46556, USA; ^3^Institute of Combustion Problems, Almaty 050012, Kazakhstan

## Abstract

A novel approach for depositing hydroxyapatite (HA) films on titanium substrates by using mechanical alloying (MA) technique has been developed. However, it was shown that one-hour heat treatment at 800°C of such mechanically coated HA layer leads to partial transformation of desired HA phase to beta-tri-calcium phosphate (**β**-TCP) phase. It appears that the grain boundary and interface defects formed during MA promote this transformation. It was discovered that doping HA by silicon results in hindering this phase transformation process. The Si-doped HA does not show phase transition to **β**-TCP or decomposition after heat treatment even at 900°C.

## 1. Introduction

Natural bone is a nanocomposite that consists of mineral fraction, including small apatite crystals and nonstoichiometric calcium phosphate, and organic fraction, which together confer mechanical resistance. In order to simulate the nature bone structure, the synthesis of hydroxyapatite (HA: Ca_10_(PO_4_)_6_(OH)_2_) has received attention in recent years. However, the HA coatings of metallic implants often flake off as a result of poor ceramic/metal interface bonding, which may cause surgery to fail [1]. This problem may be solved by fabrication of metal/HA composites. Some work has been reported on the preparation of Ti-based alloy/HA composite materials or composite coatings for biomedical applications [2–4]. But most synthetic apatites are formed via high temperature and complicate processes, resulting in a well-crystallized structure and a larger particle size, which have little or no activity in bioresorption. Since kinetic solubility is dependent on particle size, there has been great interest in nanosized HA-based cement as bone substitute materials [5, 6]. Our group also has recently described the effect of high energy ball milling treatment on HA and Ti substrate [7, 8]. It was shown that, during this type of mechanical treatment, a significant decrease of the HAs' particle and crystalline size takes place which leads to formation of nanoscaled coating on the Ti substrate. For example, it was demonstrated that one hour of such mechanical treatment with optimum ball to powder ratio equals to 40 : 1 leads to the reduction of surface particle size from 1.0 *μ*m to 80 nm.

Recently [9, 10], experimental work on HA and its substituted counterparts such as silicon-doped HA (SiHA) has focused on finding a measurable indicator of biocompatibility. It was found that dissolution or mineralization of HA may start from dislocations and defects at the grain boundaries in dental HA. Within synthetically produced hydroxyapatite, these boundaries are “clean” with few defects or voids. More close investigation of the grain boundary structures in hydroxyapatite and silicon-substituted hydroxyapatite revealed that while there was no significant difference in dislocation density between HA and SiHA there was certainly an increase in triple junctions within creased silicon doping. Also it is shown that the dissolution does occur preferentially from grain boundaries and triple junctions. Additionally, at triple junctions, it was the smallest grains that showed the greatest dissolution, suggesting that a decrease in grain size would lead to increased solubility and hence greater biocompatibility. It is of particular interest to note that after sintering, SiHA has been shown to have a much finer grain structure than pure HA-phase, suggesting that silicon also inhibits grain growth. Thus SiHA, can be considered as an important candidate as an enhanced bioactive material.

The objective of the present work is to develop high-energy ball milling approach for one-step production of nanosized SiHA coating on the surface of titanium substrate. Special attention is paid on the influence of silicon doping on the microstructure of the produced coatings.

## 2. Materials and Methods

The following procedure was used to prepare hydroxyapatite/silicon coatings on the titanium substrate. The mixtures of the hydroxyapatite (HA) (Wako, 99.5%, 250 *μ*m) and silicon (Si) powders, as well as Ti substrate (plate with dimensions 10 × 8 × 2 mm) are placed into the vibration chamber and undergo the high-energy ball milling (HEBM) with different mixture to ball weight ratios (*W*_*p*_ : *W*_*b*_). The milling process was carried out in static air without any process control agent. During HEBM, two main processes contribute for the formation of coated layer on the Ti substrate (i) fracturing of the HA and Si particles, (ii) cold welding of the mixture and its adhesion to the metal (Ti) surface. As-prepared (coated) samples were then annealed in vacuum of 10^−5^ Pa, for 1 hour, at temperatures ranging between 600°C and 1000°C.

The microstructure of the coatings was analyzed by using different material science techniques. XRD analysis was performed using DRON-6 system, by using Cu K_*α*_ radiation (wavelength *λ* = 0.15406 nm) with a nickel filter at 25 kV and 25 mA. The diffractometer was operated within range of 20 < 2*θ* < 60 with steptime: 3 seconds and stepsize: 0.02 degree. The microstructural features of the surface layer and coating distribution on substrate surface have been systematically investigated using a Solver PRO scanning probe microscope JSPM-5200 (JEOL) and scanning electron microscopy Quanta 3D 200i. The sample composition was analyzed by energy dispersive x-ray spectroscopy (EDS) using JEOL JSM-6490 LA analytical SEM. A Perkin-Elmer Optima 2000 Dual View inductively coupled plasma (ICP) optical emission spectrometer (USA) was used to carry out the chemical analysis of the used powders. Transmission electron microscopy was performed with JEOL JEM CX at an accelerating voltage of 100 kV.

## 3. Results and Discussion

### 3.1. Mechanical Milling of Hydroxyapatite Powder

**Figure 1:**
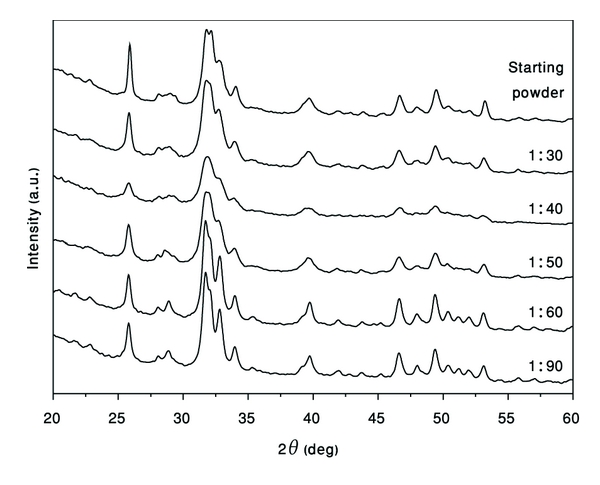
XRD patterns of hydroxyapatite powder milled one hour for various *W*_*p*_ : *W*_*b*_ ratio.

The function of HA in all of its applications is largely determined by its morphology, composition, crystal structure, and crystal size distribution. Thus, to control the mechanical properties of hydroxyapatite, the influence of synthesis conditions on such characteristics as particles' morphology and size distribution, as well as agglomeration have to be studied [11]. It is known that, for high-energy ball milling process, the weight ratio between powder mixture and milling media (balls) is one of the most important parameter, which affects the final powder microstructure.  Figure 1 shows the XRD of the HEBM-HA powders processed for one hour with different powder/ball weight ratios (*W*_*p*_ : *W*_*b*_). It can be seen that powders after milling under conditions with *W*_*p*_ : *W*_*b*_ ratio 1 : 30 and 1 : 40 are characterized by XRD patterns with notable broader peaks with lower intensity, which indicates the decreasing of phase crystalline size and powder amorphization. This result can be explained by corresponding increase in the kinetic energy of interaction between mill media and the powders [12, 13]. However, further increase of the weight of the mill media (e.g., *W*_*p*_ : *W*_*b*_ = 1 : 60; 1 : 90) leads to the recrystallization of HA powder. The latter effect may be related to the extremely high kinetic energy of the ball mill charge which transforms into heat, thus, promoting the recrystallization process.

Using Scherrer equation, the unit cell dimensions were calculated for the 1 : 60 and 1 : 90 samples (Table 1). The slight variation of lattice parameters could be due to strain accumulated through the HEBM process. Indeed, since large plastic deformation is induced into the powder particles during mechanical milling, the crystals are strained, and the deformation occurs in an inhomogeneous manner. From the XRD data, it can also be concluded that, decomposition of HA phase or formation of secondary phases, such as tricalcium phosphate, tetracalcium phosphate, and calcium oxide do not take place throughout the milling process.

### 3.2. Hydroxyapatite Powder Morphology

**Figure 2:**
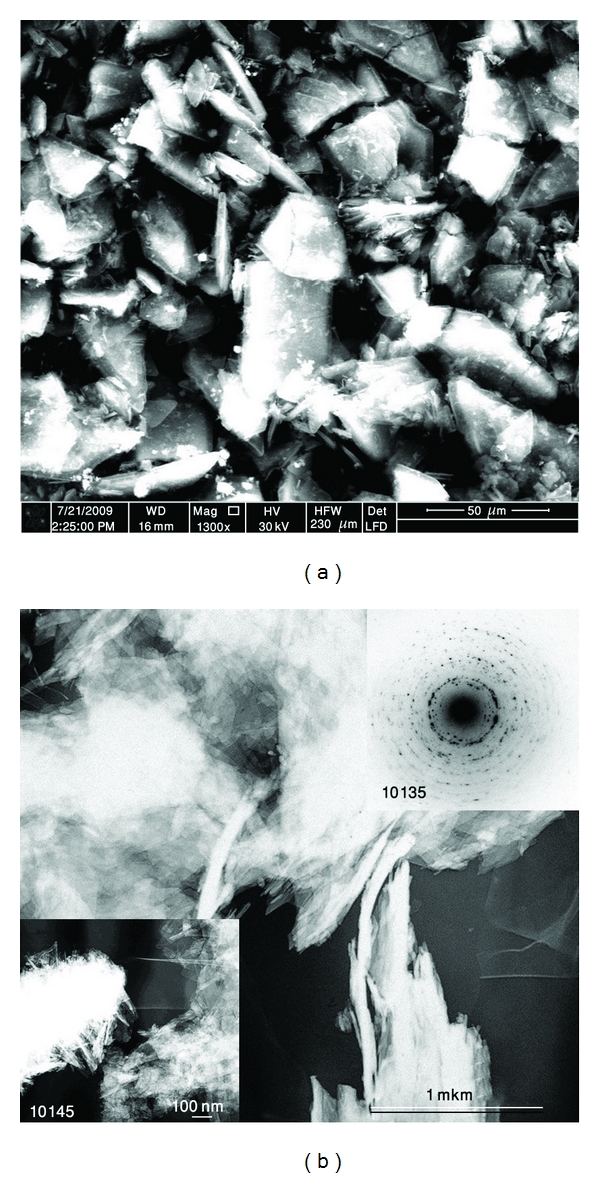
Needle-like crystallite of HA sample before milling (a) SEM and (b) TEM, respectively.

**Figure 3:**
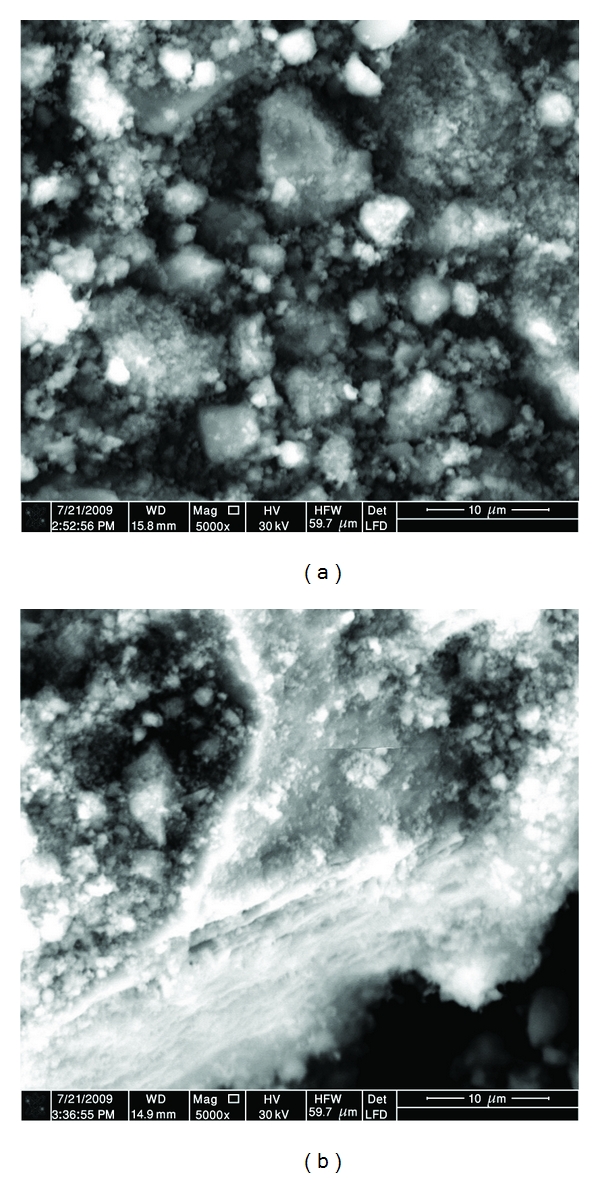
(a) and (b) Morphologies of HA powder particles after one hour of milling with *W*_*p*_ : *W*_*b*_ = 1 : 40.

Scanning electron microscopy (SEM) and transmission electron microscopy (TEM) were used to analyze the morphology of the HA powders after high-energy ball milling. As mentioned in the above section, cold welding and fracturing are the two essential processes involved in the HEBM process. Fracture tends to break individual particles into smaller pieces and deagglomerates particles that have been cold welded. The morphologies of the HA powder particles before ball milling are shown in Figure 2. It can be seen that HA powder consists of crystallites which have needles and platelets-like morphologies. Higher magnification shows that large HA particles composed of very smaller ones with the size of less than 100 nm. After 1 hour of HEBM (*W*_*p*_ : *W*_*b*_ = 1 : 40), the morphology of the initial large particles significantly changes (Figure 3) due to microforging, fracture, agglomeration, and deagglomeration processes. Thin layered (laminated) microstructure dominates in this sample.

**Figure 4:**
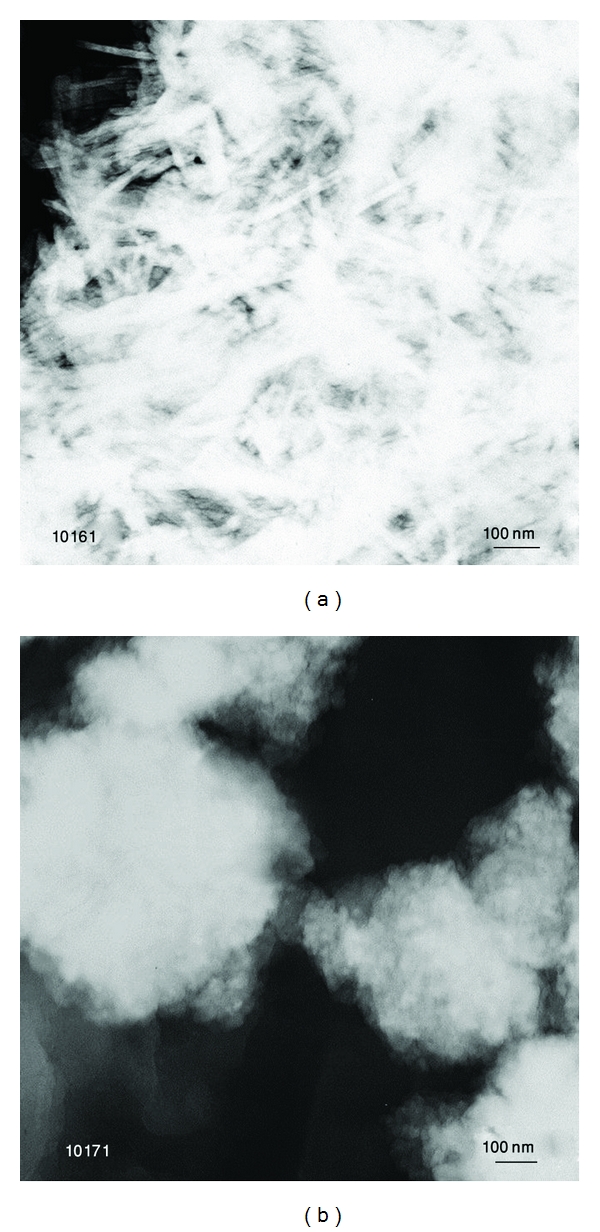
Hydroxyapatite powder after one hour of milling with *W*_*p*_ : *W*_*b*_ = 1 : 40.

However, more close inspection by TEM (Figure 4) shows two types of morphologies, that is, needle-like (a) and round shaped (b). It is also can be seen that these small particles formed the agglomerates as a result of cold welding phenomenon during ball milling.

Mechanical, physical, and chemical properties of powders may be altered if they are contaminated. The most common contaminations in mechanical milling are Fe and Cr elements that come from the milling tools (vial and balls) since most of them are made from those types of elements. The Fe and Cr elements content after one hour of HEBM is shown in Table 2. As the *W*_*b*_ : *W*_*p*_ ratio increases the amount of contamination increases. It was shown that, for the lower ratio (e.g., 10 : 1), a thin coating of the milling balls by the HA powder is formed which reduces Fe and Cr contamination. For higher *W*_*b*_ : *W*_*p*_ ratio (60 : 1), the number of collisions and friction increases, which lead to a significant increase in Fe and Cr contamination.

### 3.3. X-Ray Diffraction of HA Coating on Ti-Substrate

**Figure 5:**
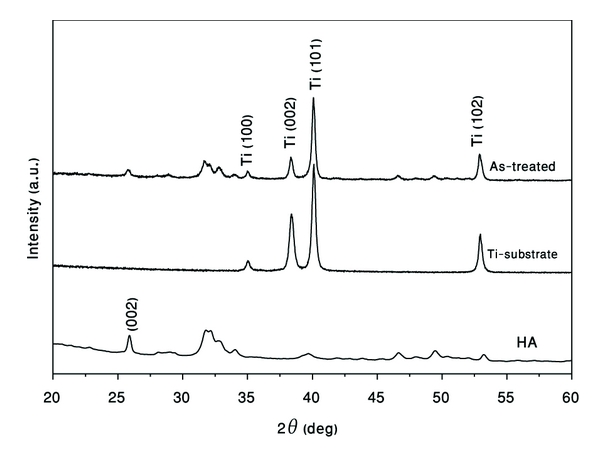
XRD patterns of ball-milled hydroxyapatite-coated Ti substrate after one hour of milling.

The XRD patterns shown in Figure 5 illustrate the effect of mechanical treatment on Ti substrate milled with HA powder. After 1 hour of HEBM, the (002) peak which is the most distinct reflection in the XRD pattern for HA possesses a notable intensity reduction. The (101) peak of the Ti substrate only slightly shifted to higher values, Δ*d* = −0.0024 and also (002) and (102) peaks are shifted with Δ*d* = −0.0015 and Δ*d* = −0.0016, respectively.

As reported [6], the process of covering metallic surfaces with HA at elevated temperatures (e.g., plasma spraying) has a tendency to eliminate the functional group OH in the HA matrix (dehydration) and results in the decomposition of HA into *α*-tricalcium phosphate, *β*-tricalcium phosphate, and tetracalcium phosphate. Also the condition of high substrate temperature promoted the oxidation of the substrate surface prior to the growth of the HA layer. The oxidation layer degraded the adhesion of the coating to the substrate. As can be concluded from XRD patterns in Figure 5, the repeated ball collision with the Ti substrate resulted in the deposition of HA powder on its surface without any trace of HA decomposition or Ti oxidation.

### 3.4. Morphology of HA Coating on Ti Substrate

**Figure 6:**
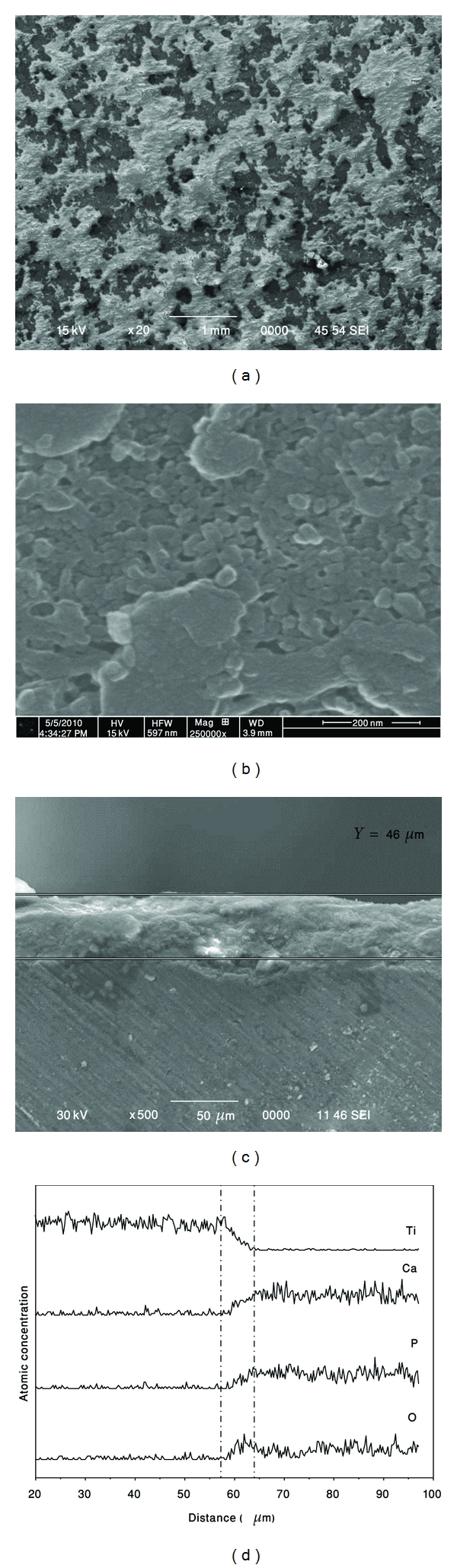
(a) and (b) Surface morphologies of the as-synthesized HA coating after one hour of milling (c) cross-section microstructure and (d) concentration profile of as-synthesized HA coating.

Typical morphology of the coated substrate surface is shown in Figure 6. It can be seen that after one hour of HEBM, the substrate was covered with HA powder. The inhomogeneous distribution over the entire coated sample could be due to powder particles repeatedly fractured and cold welded on the substrate. The similar result, that is, inhomogeneous distributed precipitates was observed for calcium and phosphorus ion implanted in a dose of 10^17^ ions/cm^2^ [14]_. _The broad face morphology of the as-coated HA (Figure 6(b)) implies that the agglomeration of particles occurs due to cold welding process. The dome-shaped morphology was also formed in physical-vapor-deposited and laser-deposited HA films [15].

Typical cross-sections of as-treated HA coating is shown in Figure 6(c) where the coating thickness was about 50 *μ*m. The composition of the HA-coated sample was analyzed by energy dispersive X-ray spectroscopy (EDS).  Figure 6(d) shows the cross-section microstructure and concentration profile of HA coating produced by mechanical treatment method. Cold welding between particles and substrate under repeated ball collisions led to the formation of a composite coating. HA (Ca, P, O-elements) flowed into the pores between Ti particles under the impact of balls and vice versa. The EDS analysis of the as-treated sample indicated that the average value of the Ca/P ratio of coated HA was (1.803 ± 0.18) while the stochiometric molar ratio is 1.67 [16].

**Figure 7:**
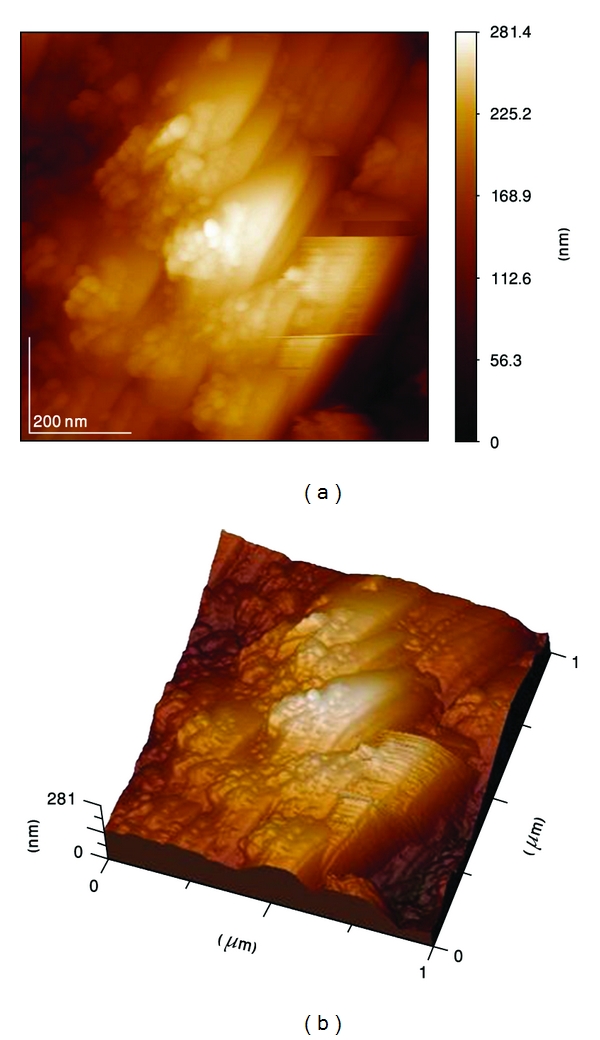
2-dimension and 3D AFM image of the as-coated HA.

**Figure 8:**
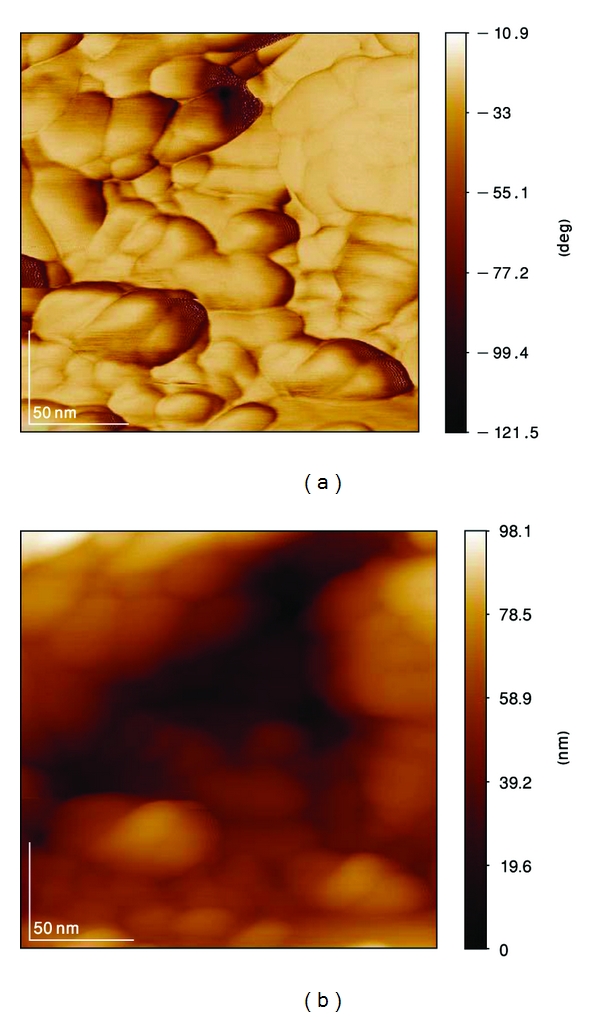
Phase and topography of as-coated HA.

Atomic force microscopy (AFM) image of the as-coated HA, Figure 7 showed that the HA was composed of numerous spherical-shaped aggregates of different sizes. The higher magnification micrograph, Figure 8, revealed that each spheroid-type aggregate involves a large number of much smaller grains.

### 3.5. X-Ray Diffraction of the Annealed HA Coating on Ti-Substrate

**Figure 9:**
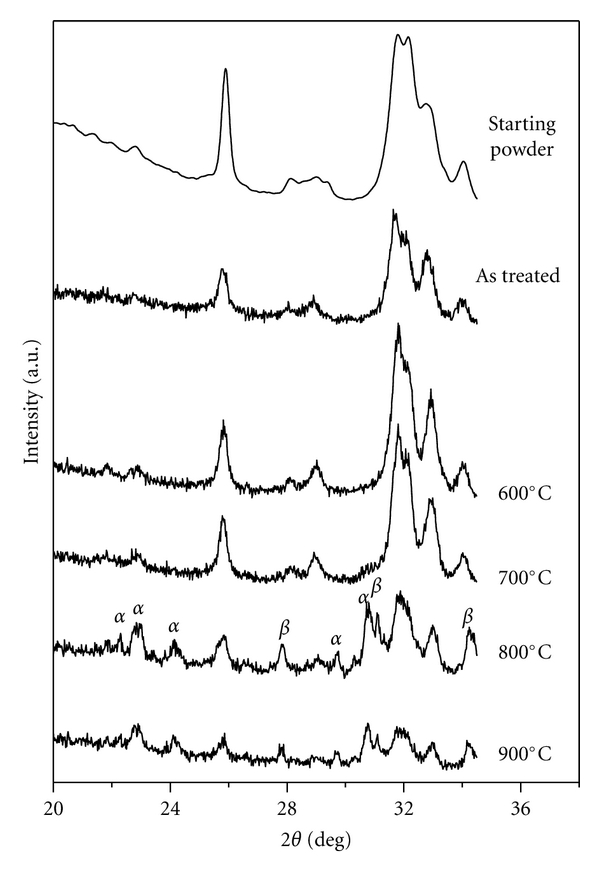
XRD patterns of HA deposited on Ti substrate after heated at various temperatures.

XRD patterns of deposited HA heated at different temperatures are presented in Figure 9. X-ray peaks of the formed phase were matched to the ICDD (JCPDS) standard, *α*-tricalcium phosphate (09–0348), *β*-tricalcium phosphate (03–0690), and titanium oxide (29–1361). The changes in the XRD patterns give an indication of the influence of temperature on the structure stability of the samples. According to XRD patterns of the coated samples, it is clear that up to 700°C HA phase is stable; that is, no any phase transition or decompositions were observed. With increasing heat treatment, temperature the intensity of HA peaks increases as compared to that of as-treated sample. As the heat treatment temperature raised to 800°C, the slight decomposition of the HA to Ca_3_(PO_4_)_2_ (TCP) can be observed. The amount of TCP increases after 900°C.

The relatively low (800°C) decomposition temperature is could be due to diffusivity enhancement in HA because of Ti presence and/or effect of MA. It is reported [17] that the HA thermal decomposition occurs in two steps: dehydroxylation and decomposition. Dehydroxylation to oxyhydroxyapatite proceeds at temperatures in the range 850–900°C. The decomposition to TCP and TTCP occurs at temperatures greater than 900°C.

 Also, since both the dehydroxylation and decomposition reactions include water vapor as a product, the rates at which these reactions proceed depend on the partial pressure of H_2_O in the furnace. Therefore, the secondary phase formation during sintering could be suppressed by controlling the moisture content in the sintering atmosphere. The high moisture content has the tendency to slow down the decomposition rate by preventing the dehydration of the OH group from the HA matrix. The difference in result in the present work with others could in part be also attributed to the difference in humidity in the sintering atmosphere and also to the nature of the deposited material (mechanical treated samples). Finally, the transformation from HAP to *β*-TCP phase may occurr at relatively low temperature because of the nanosize nature of HEBM-HAP coating, since fine particles are less stable for heat treatment.

According to [18], heat treatment of HA at 800°C for 5 h was sufficient for HA to *β*-TCP transformation. Also, it was shown [19] that, for Ti-doped samples (100 and 200 ppm of Ti), an endothermic peak, which corresponds to the HA→ *β*-TCP transformation, occurs at 794°C. Note that no reactions were observed in the pure and 50 ppm Ti-doped HA samples. The formation of *β*-TCP is also a sensitive indicator for Ca/P ratio in HA composition.

Thus, the most likely explanation for the existence of *β*-TCP phase in the heated HEBM-synthesized HA-Ti sample is that the presence of Ti in the incubation media leads to the formation of a calcium-deficient apatite that has a Ca/P ratio less than 1.67. Calcium-deficient HA is less thermally stable than stoichiometric HA. The enhanced diffusivity is believed to be due to the introduction of structural defects such as grain boundaries and interfaces. The correlation between the enhanced diffusivity and the composite microstructure is often reflected by the decrease of reaction temperature with refinement of the composite microstructure. It appears that the interface plays a major role in reducing the reaction temperature. With the reduction of layer thickness and the increase of interface area, the reaction becomes easier. When the reaction becomes easy enough, it can occur during milling, leading to mechanical alloying [20].

### 3.6. Morphology of the Annealed HA Coating on Ti Substrate

**Figure 10:**
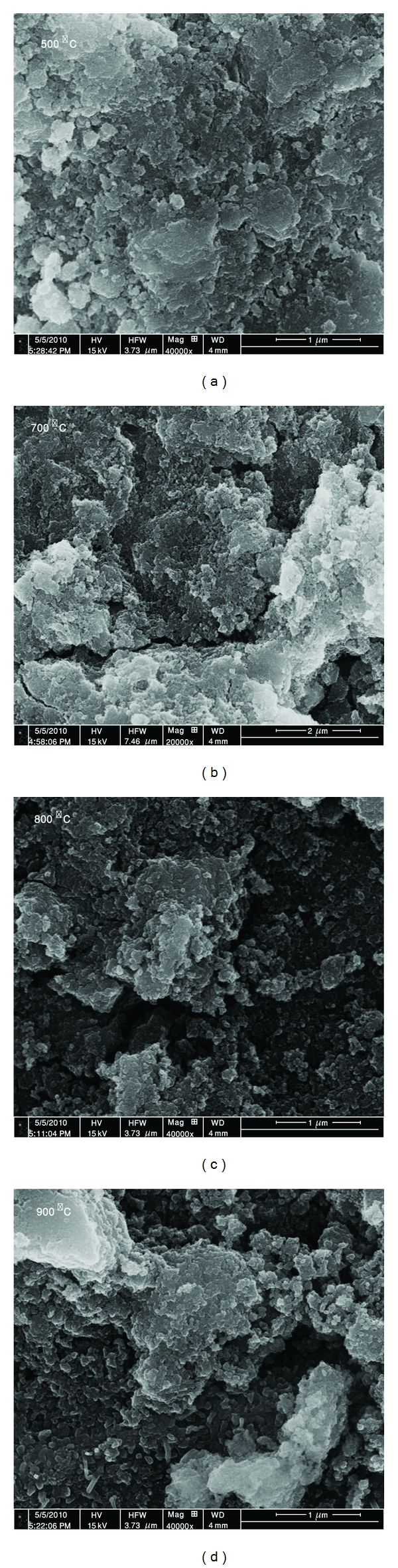
SEM of heat-treated hydroxyapatite coatings.

A set of backscattered electron images is shown in Figure 10, which illustrate the microstructure evolution of the HA surface during samples heat treatment. It can be seen that cracks are formed, while no any other microstructural changes can be detected. The presence of the cracks could be due to the difference in coefficients of thermal expansion of HA and Ti-alloy substrate. According to linear elastic fracture mechanics, a constrained film, subjected to stress, would crack when the strain energy released in the process exceeds the energy required to form the crack [21]. Also, cracks could form during cooling of annealed ceramic films deposited on a substrate due to differences between the thermal expansion coefficients of the substrate and the coated film. Finally, MA is an effective way of creating localized plastic deformation, so the cracks formation after heat treated also could be due to mechanical energy released.

**Figure 11:**
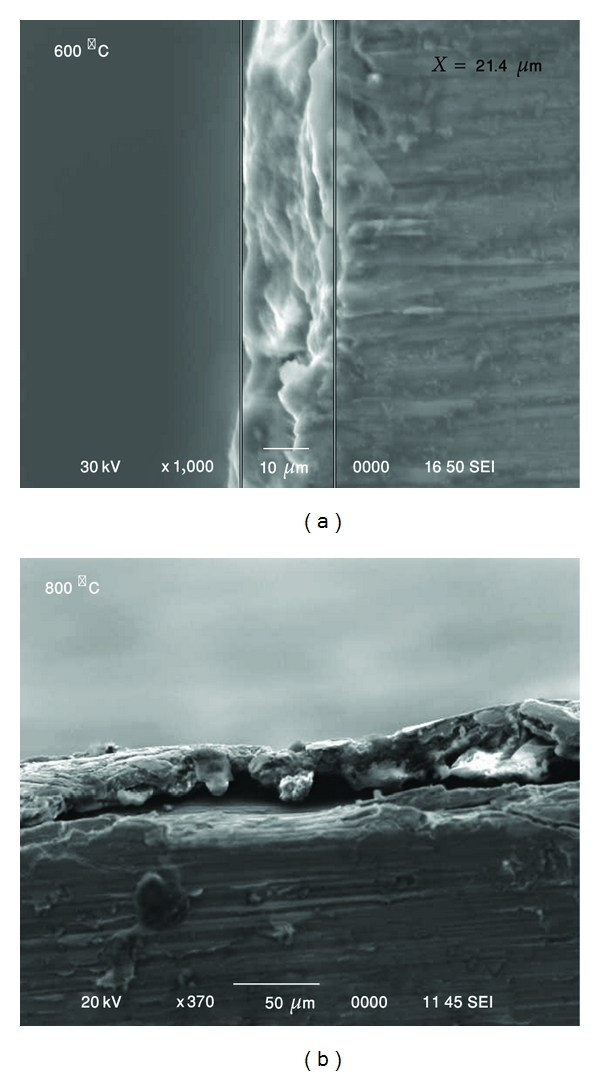
Cross-section microstructure of heat-treated HAe layer.

Figure 11 illustrates a typical SEM view of cross-sections for samples heat treated under different temperatures. The multilayer coatings can be readily seen after heat treatment at 600°C (Figure 11(a)), which could be due to repeated fracture. The coating layer was partially dissociated after 800°C (Figure 11(b)).

**Figure 12:**
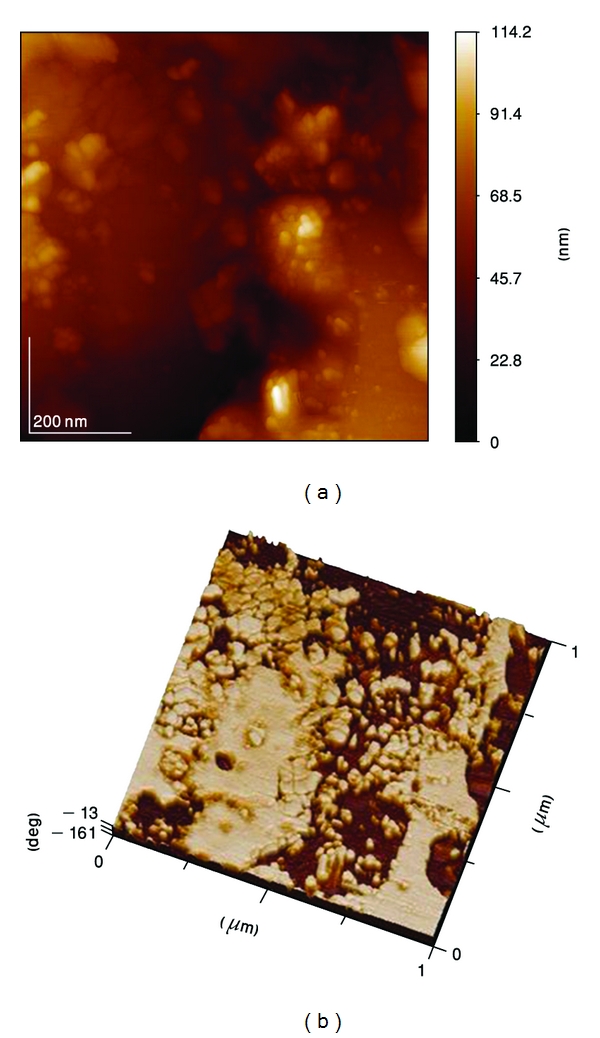
Surface structure analyzed by means of AFM of heat-treated hydroxyapatite at 700°C. (a) Topography and (b) Phase.

AFM observation of the coating (Figure 12) showed the presence of nanosized HA. This feature could not be resolved by SEM. The high specific area generated by this topography may be associated to the high bioactivity of this coating. According to the AFM measurements, the HA range of particle sizes for the annealed sample at 700°C is 40–70 nm.

### 3.7. X-Ray Diffraction Silicon-Doped Hydroxyapatite

**Figure 13:**
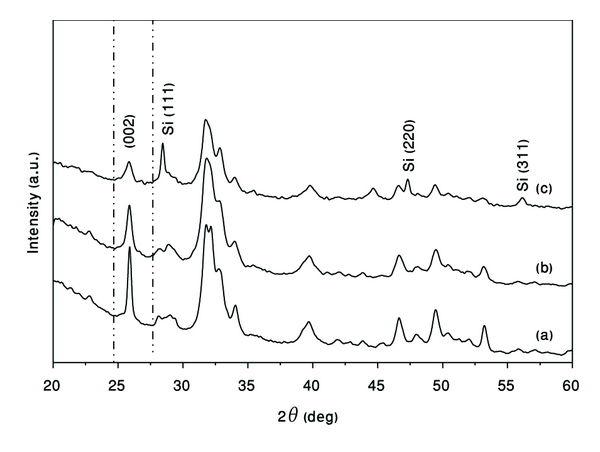
XRD patterns of (a) hydroxyapatite powder, (b) milled hydroxyapatite, and (c) mechanical alloying Si-doped hydroxyapatite.

Figures 13(a) and 13(b) shows the XRD patterns of the pure HA powder before and after milling for 1 h, while Figure 13(c) show such patterns for Si-doped HA powder. Significant changes were detected in the XRD patterns when after HEBM. The Si-doped sample shows a notable broadening and intensity reduction as compared to pure HA powders before and after milling. Thus, the phase crystallinity strongly decreases when silicon enters into the HA structure. The same behavior was observed in [22] with Si content less than 2.41 weight percent. Due to the mechanical deformation introduced into the powder during HEBM and because Si enhances solubility, the particle and crystallite for HA phase could be refined.

Also, the (002) peak for SiHA sample (Figure 13) shows a notable broadening and intensity reduction comparing with pure HA. The wide peaks of the deposited materials should be due to very small crystallite size and microstrain. Issues about crystal structure changes in the HA with incorporation of silicon have been addressed by several experimental groups. Importantly it has been found that the incorporation of silicon into the crystal has little effect on the crystal structure. X-ray diffraction studies revealed that the intensity, width, and position of peaks for 0.4, 0.8, and 1.5 wt% silicon HA were very similar to those of phase-pure hydroxyapatite. Although there are no dramatic changes in the crystal structure, it should be noted that increasing the silicon content does produce a change in HA lattice parameters. Recent experimental work has shown that there is a systematic increase in the c-lattice parameter and a concomitant increase in cell volume with increasing silicon content. It is important to recognize that the silicate has a formal charge of −4, compared to −3 for the phosphate group. It was experimentally found that, for compensating of this excess of a negative charge, one hydroxyl group leaves the crystal for every silicon substitution [23].

### 3.8. Morphology of Silicon-Doped Hydroxyapatite

**Figure 14:**
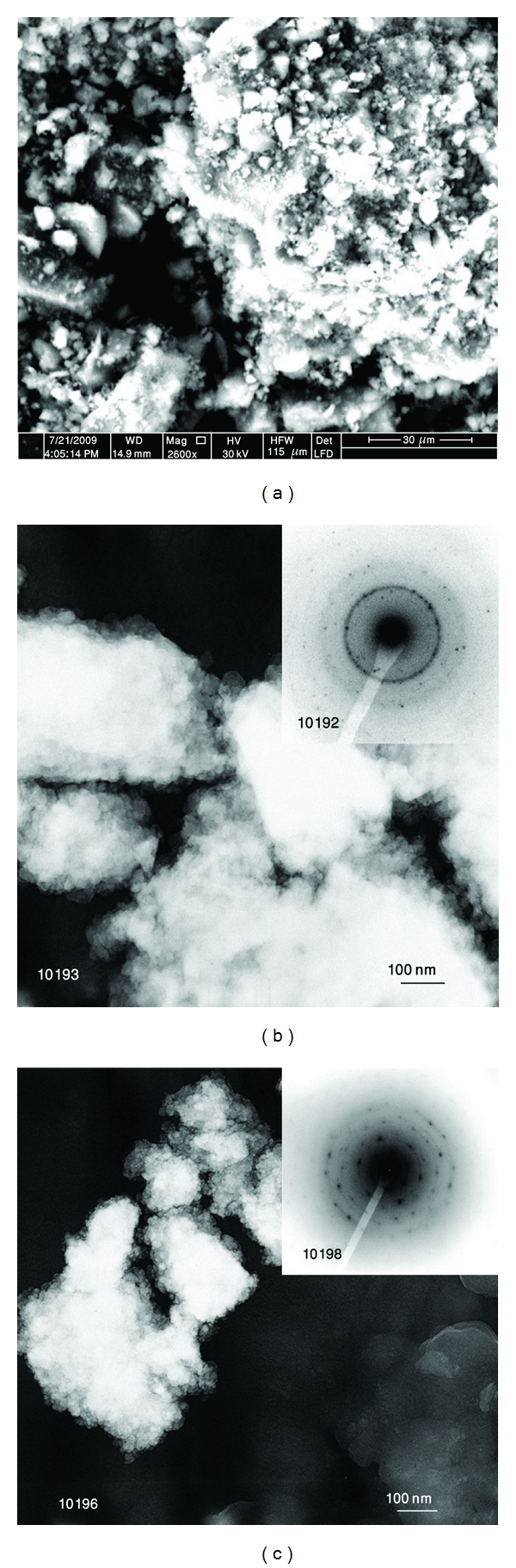
SEM (a) and TEM ((b), (c)) images Si-doped HA after 1 h of HEBM.

Morphology of the Si-dope HA powder was examined using SEM and TEM techniques. Cold welding and fracturing are the two essential processes involved in the mechanical milling/alloying process. After HEBM (*W*_*p*_ : *W*_*b*_ = 1 : 40) the particles morphology changes due to microforging, fracture, agglomeration, and deagglomeration. Thus the particle becomes smaller in size due to fracturing and agglomerated by cold welding through the milling process (Figure 14(a)). The TEM pictures of mechanically milled Si-dope HA sample are presented in Figures 14(b) and 14(c). It can be seen that a small round-shaped crystals with size of ~25 nm are formed during milling process.

### 3.9. X-Ray Diffraction of Si-Doped HA Coating

**Figure 15:**
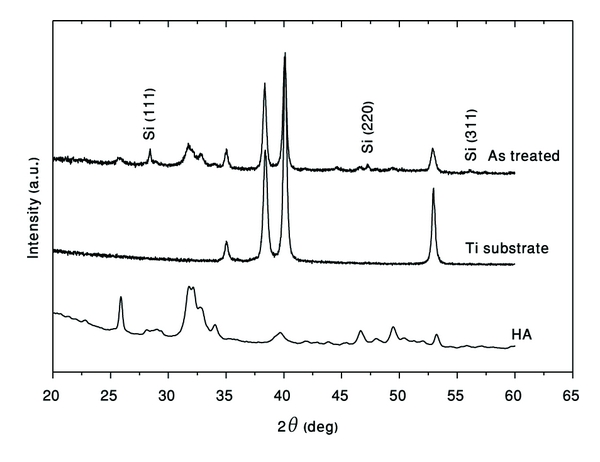
XRD patterns of the SiHA-coated Ti substrate.

XRD patterns of deposited HA and SiHA are presented in Figure 15. Coated SiHA on Ti substrate demonstrates the foundation of HA, as well as Si peaks. Comparison of these patterns with those obtained in [7, 8] shows the influence of high-energy ball milling process on the structure stability of the formed phases.

### 3.10. Morphology of Si-Doped HA Coating

**Figure 16:**
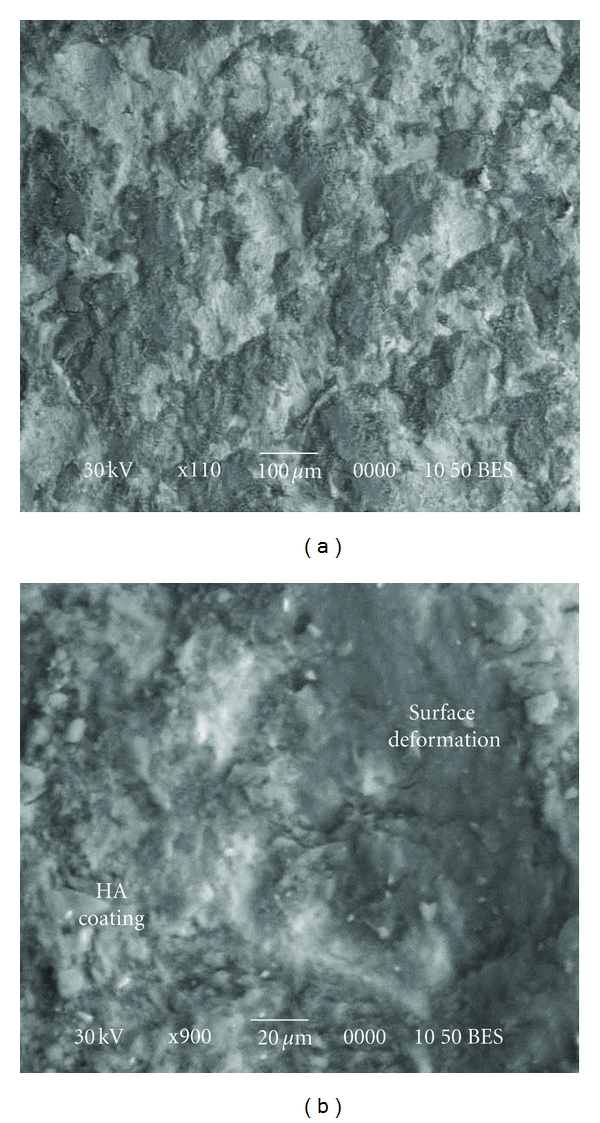
Backscattered electron images of the as-synthesized SiHA coating after one hour of mechanical treatment.

**Figure 17:**
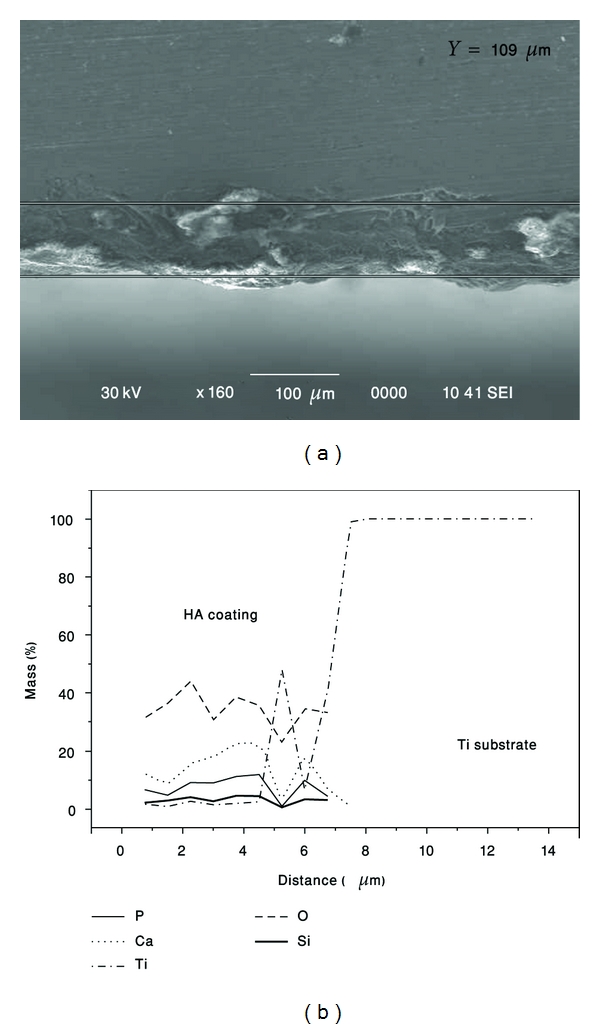
Structure of as-synthesized SiHA coating produced by MA method: (a) cross-section microstructure, (b) concentration profile.

Backscattered electron images of the Si-doped HA surfaces “deposited” on Ti-alloy substrate are shown in Figure 16. After 1 hour of HEBM, the substrate was covered by HA layer in much more extent as compared to pure HA powder. This could be due to Si addition which improves the adhesion of HA to substrate. At higher magnification (Figure 16(b)), one can see the Ti-surface deformation and HA adhesion to the substrate.  Figure 17(a) shows the microstructure of the cross-section of the Si-doped HA coating. The composition along the coated sample was analyzed by energy dispersive X-ray spectroscopy (EDS).  Figure 17(b) shows the corresponding concentration profile at the boundary between the intermixing region and the coating. It can be concluded that cold welding between particles and substrate under repeated ball collisions led to the formation of a composite coating where the HA (Ca, P, and O elements) and Si phases flowed into the pores in Ti substrate. The homogeneous distribution of Si particles in the HA formed by HEBM improves the adhesion between coating and substrate.

### 3.11. X-Ray Diffraction of Heat Treatment Si-Doped HA Coating

**Figure 18:**
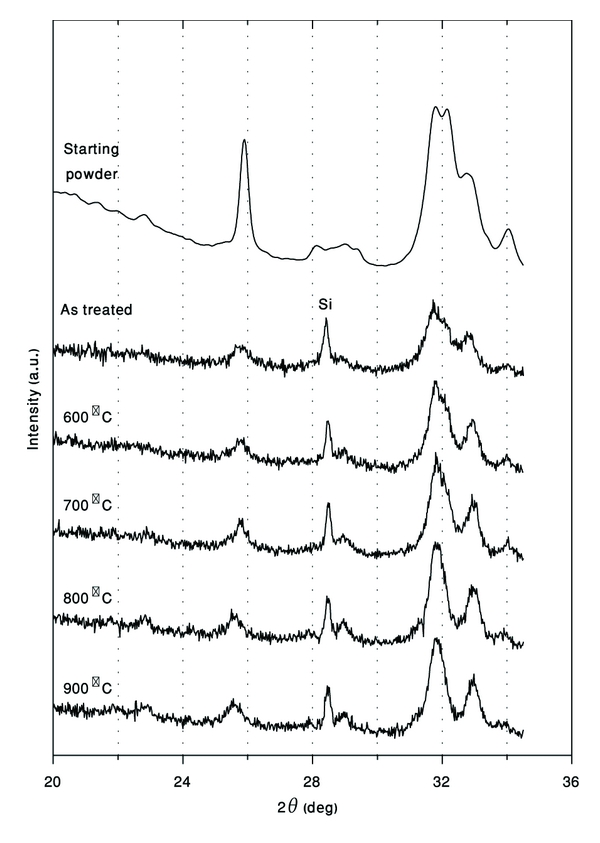
XRD patterns of SiHA deposited on Ti substrate after being heated at various temperatures (all peaks, except of shown Si peak, belong to HA phase).

XRD patterns of Si-doped HA heat treated at different temperatures are presented in Figure 18. The changes in the XRD patterns give an indication of the influence of temperature on the structure stability of the samples. X-ray peaks of the formed phase were matched to the ICDD (JCPDS) standard. XRD pattern of the as-treated sample shows wide peaks owing to very small size of formed crystallites. After heat treatment, peaks shifted slightly to higher diffraction angles due to strain relaxation. It is important that in the whole range of heat treatment conditions (up to 900°C), Si-doped HA samples have not shown phase transition or decomposition. With increasing the heat treatment temperature, only the intensity of HA peaks increases due to recrystallization process.

### 3.12. Morphology of Heat Treatment Si-Doped HA Coating

**Figure 19:**
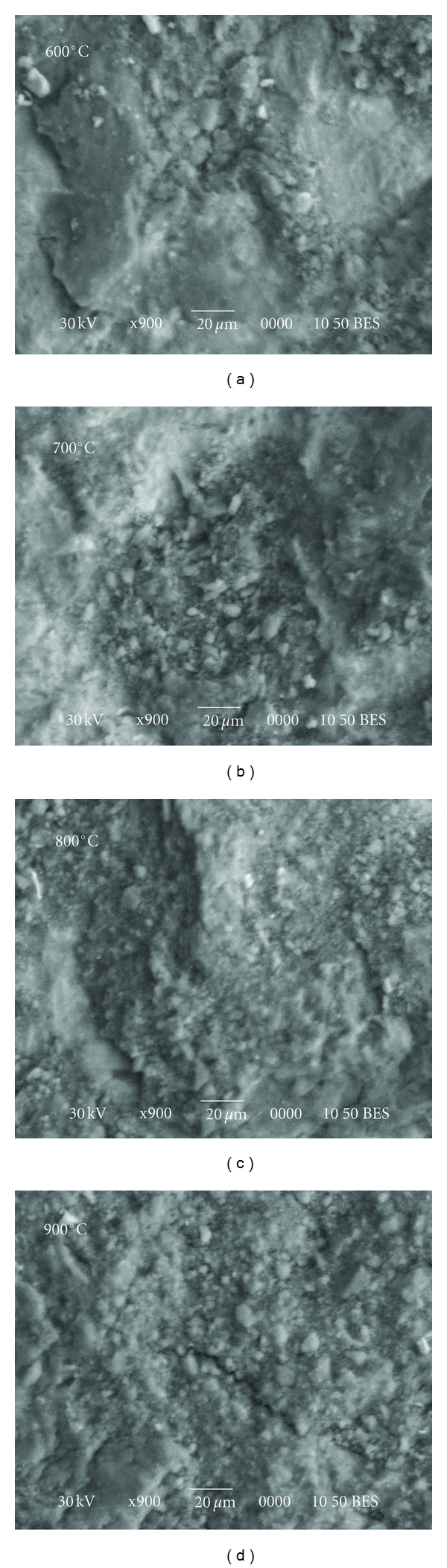
Backscattered electron images of the heat-treated SiHA coating by MA.

Figure 19 shows set of backscattered electron images, indicating the microstructures of coating surfaces obtained under various heat treatment conditions. It is worth noting that no any visible changes in microstructure were observed after treatment in the range of 600–800°C. The cracks are detected only after treatment at high temperature of ~900°C. However, the interface between HA and a metallic implant has been another matter of concern in terms of the mechanical properties and biocompatibility of the implant. In order for the HA coating to be effective and reliable, it must be strongly bonded to the metallic surface. The analysis of cross-section of heat-treated Si-doped HA samples (thickness 130 *μ*m at 700°C and 55 *μ*m at 900°C) shows that the coatings possess good adhesion to the Ti substrates.

## 4. Conclusion

Thus, based on the detailed studies of using the high-energy ball milling technique for depositing ceramic coating on the metal substrate, a novel approach for production of hydroxyapatite (HA) films on titanium substrates has been developed. During HEBM process, the impacts of the milling balls activate the metal surface and lead to the robust cold welding of HA particles to the metal surface. It was shown that HEBM also results in significant decrease of the HA particle and crystalline size, forming the nanoscale structure. It was demonstrated that a heat treatment of the mechanically coated HA at 800°C for one hour leads to partial transformation of HA phase to *β*-TCP. It appears that the grain boundary and interface defects formed during HEBM reduce the transformation temperature. Also, it was shown that Ti incorporation into the HA structure not only causes the lattice shrinkage and reduction of its grain size as compared to pure HA, but also promotes the phase transformation of HA to TCP during heat treatment. It is important that doping HA by silicon, while significantly decreases the crystal size of HA layer, it results in hindering of the phase transformation process. The Si-doped HA does not show phase transition or decomposition after heat treatment even at 900°C.

## Figures and Tables

**Table 1:** Unit cell parameters of the mechanical milled HA.

Sample	Lattice constant (Å)	Standard deviation	*R*_*wp*_ (%)
Standard card (24–0033)	*a* = *b* = 9.432 *c* = 6.881	—	
1 : 60 ratio	*a* = *b* = 9.443 *c* = 6.898	0.0014	7.6
1 : 90 ratio	*a* = *b* = 9.430 *c* = 6.898	0.0018	8.9

*R*_*wp*  (weighted  residual  error)_.

**Table 2:** Fe and Cr contamination in one-hour milled HA powder.

	Elements			
	Fe		Cr	
*W*_*b*_ : *W*_*p*_ ratios	ppmw	%	ppmw	%
Before milling	32.3 ± 1.9	0.0032	<1	<10^−4^
10 : 1	302 ± 9	0.03	3.3 ± 0.2	3.3 × 10^−4^
30 : 1	480 ± 14	0.048	6.2 ± 0.3	6.2 × 10^−4^
60 : 1	4787 ± 144	0.479	60 ± 2	6.0 × 10^−3^

## References

[1] Johnson S, Haluska M, Narayan RJ, Snyder RL (2006). In situ annealing of hydroxyapatite thin films. *Materials Science and Engineering C*.

[2] Albayrak O, El-Atwani O, Altintas S (2008). Hydroxyapatite coating on titanium substrate by electrophoretic deposition method: effects of titanium dioxide inner layer on adhesion strength and hydroxyapatite decomposition. *Surface and Coatings Technology*.

[3] Khor KA, Yip CS, Cheang P (1997). Ti-6Al-4V/hydroxyapatite composite coatings prepared by thermal spray techniques. *Journal of Thermal Spray Technology*.

[4] Wang CK, Chern Lin JH, Ju CP, Ong HC, Chang RPH (1997). Structural characterization of pulsed laser-deposited hydroxyapatite film on titanium substrate. *Biomaterials*.

[5] Xiao Y, Li D, Fan H, Li X, Gu Z, Zhang X (2007). Preparation of nano-HA/PLA composite by modified-PLA for controlling the growth of HA crystals. *Materials Letters*.

[6] Shih WJ, Chen YF, Wang MC, Hon MH (2004). Crystal growth and morphology of the nano-sized hydroxyapatite powders synthesized from CaHPO_4_
*·*2H_2_O and CaCO_3_ by hydrolysis method. *Journal of Crystal Growth*.

[7] Hannora A, Mamaeva A, Mansurov Z (2009). X-ray investigation of Ti-doped hydroxyapatite coating by mechanical alloying. *Surface Review and Letters*.

[8] Hannora A, Mamaeva A, Mofa N, Aknazarov S, Mansurov Z (2009). Formation of hydroxyapatite coating by mechanical alloying method. *Eurasian Chemico-Technological Journal*.

[9] Lopez-A lvarez M, Solla EL, Gonzalez P (2009). Silicon-hydroxyapatite bioactive coatings (Si-HA) from diatomaceous earth and silica. Study of adhesion and proliferation of osteoblast-like cells. *Journal of Materials Science: Materials in Medicine*.

[10] Zou S, Huang J, Best S, Bonfield W (2005). Crystal imperfection studies of pure and silicon substituted hydroxyapatite using Raman and XRD. *Journal of Materials Science: Materials in Medicine*.

[11] Shih WJ, Wang JW, Wang MC, Hon MH (2006). A study on the phase transformation of the nanosized hydroxyapatite synthesized by hydrolysis using in situ high temperature X-ray diffraction. *Materials Science and Engineering C*.

[12] Suryanarayana C (2001). Mechanical alloying and milling. *Progress in Materials Science*.

[13] El-Eskandarany MS, Aoki K, Itoh H, Suzuki K (1991). Effect of ball-to-powder weight ratio on the amorphization reaction of Al50Ta50 by ball milling. *Journal of The Less-Common Metals*.

[14] Krupa D, Baszkiewicz J, Kozubowski JA (2004). Effect of calcium and phosphorus ion implantation on the corrosion resistance and biocompatibility of titanium. *Bio-Medical Materials and Engineering*.

[15] Díaz-Estrada JR, Camps E, Escobar-Alarcón L, Ascencio JA (2007). Mechanical improvement of hydroxyapatite by TiOx nanoparticles deposition. *Journal of Materials Science*.

[16] White T, Ferraris C, Kim J, Madhavi S (2005). Apatite—an adaptive framework structure. *Reviews in Mineralogy and Geochemistry*.

[17] Ramesh S, Tan CY, Sopyan I, Hamdi M, Teng WD (2007). Consolidation of nanocrystalline hydroxyapatite powder. *Science and Technology of Advanced Materials*.

[18] Mayer I, Cuisinier FJG, Gdalya S, Popov I (2008). TEM study of the morphology of Mn^2+^-doped calcium hydroxyapatite and *β*-tricalcium phosphate. *Journal of Inorganic Biochemistry*.

[19] Ribeiro CC, Gibson I, Barbosa MA (2006). The uptake of titanium ions by hydroxyapatite particles—structural changes and possible mechanisms. *Biomaterials*.

[20] Zhang DL (2004). Processing of advanced materials using high-energy mechanical milling. *Progress in Materials Science*.

[21] Mahé M, Heintz JM, Rödel J, Reynders P (2008). Cracking of titania nanocrystalline coatings. *Journal of the European Ceramic Society*.

[22] Sprio S, Tampieri A, Landi E (2008). Physico-chemical properties and solubility behaviour of multi-substituted hydroxyapatite powders containing silicon. *Materials Science and Engineering C*.

[23] Chappell HF, Bristowe PD (2007). Density functional calculations of the properties of silicon-substituted hydroxyapatite. *Journal of Materials Science: Materials in Medicine*.

